# Health system decision-makers at the helm of implementation research: development of a framework to evaluate the processes and effectiveness of embedded approaches

**DOI:** 10.1186/s12961-020-00579-9

**Published:** 2020-06-10

**Authors:** N. Ilona Varallyay, Etienne V. Langlois, Nhan Tran, Vanesa Elias, Ludovic Reveiz

**Affiliations:** 1 Department of International Health of the Johns Hopkins Bloomberg School of Public Health, Baltimore, MD United States of America; 2 Alliance for Health Policy and Systems Research, World Health Organization, 20 Avenue Appia, 1211 Geneva, Switzerland; 3 Unintentional Injury Prevention Department for the Management of Non-communicable Diseases, Disability, Violence, and Injury Prevention (NVI), World Health Organization, 20 Avenue Appia, 1211 Geneva, Switzerland; 4 Department of Evidence and Intelligence for Action in Health, Pan American Health Organization, 525 Twenty-third Street, N.W, Washington, D.C, USA

**Keywords:** Embedded research, implementation research, evidence-informed decision-making, research co-production, evidence-to-action, knowledge translation, health policy and systems research, evaluation framework, low- and middle-income countries

## Abstract

**Background:**

Embedded approaches to implementation research (IR), whereby health system decision-makers participate actively in the research process, are gaining traction as effective approaches to optimise the delivery of health programmes and policies. However, the evidence base on the processes and effectiveness of such collaborative research remains inchoate. Standardised approaches to evaluate these initiatives are needed to identify core elements of ‘embeddedness’, unveil the underlying pathways of change, and assess contribution to evidence uptake in decision-making and overall outcomes of effect. The framework presented in this paper responds to this need, designed to guide the systematic evaluation of embedded IR.

**Methods:**

This evaluation framework for embedded IR approaches is based on the experience of a joint initiative by the Pan American Health Organization/Alliance for Health Policy and Systems Research, which has supported 19 IR grants in 10 Latin American and Caribbean countries from 2014 to 2017. The conceptualisation of this framework drew on various sources of information, including empirical evidence and conceptual insights from the literature, interviews with content experts, and a prospective evaluation of the 2016 cohort that included semi-structured key informant interviews, document analysis, and a research team survey to examine key aspects of embedded research.

**Results:**

We developed a widely applicable conceptual framework to guide the evaluation of embedded IR in various contexts. Focused on uncovering how this collaborative research approach influences programme improvement, it outlines expected processes and intermediate outcomes. It also highlights constructs with which to assess ‘embeddedness’ as well as critical contextual factors. The framework is intended to provide a structure by which to systematically examine such embedded research initiatives, proposing three key stages of evidence-informed decision-making – co-production of evidence, engagement with research, and enactment of programme changes.

**Conclusion:**

Rigorous evaluation of embedded IR is needed to build the evidence on its processes and effectiveness in influencing decision-making. The evaluation framework presented here addresses this gap with consideration of the complexity of such efforts. Its applicability to similar initiatives is bolstered by virtue of being founded on real-world experience; its potential to contribute to a nuanced understanding of embedded IR is significant.

Contributions to the literature

- Embedded implementation research (IR) is a promising approach to promote the use of research for public sector decision-making; however, the evidence base on its processes and effectiveness remains inchoate.
- Systematic evaluation is needed to advance our understanding about how embedded IR operates in real-world contexts.
- This manuscript fills a gap in the literature, presenting a comprehensive, widely applicable framework to guide such evaluations.
- The use of this framework can help advance the science and practice of embedded research — a valuable endeavour as achievement of the UN Sustainable Development Goals will depend largely on evidence-informed programmes/policies, particularly in low-resource settings.

## Background

One of the greatest challenges in the global health field and in the social development sector more broadly is the effective implementation of evidence-informed policies and practice. Implementation research (IR) specifically aims to “*improve the effectiveness, quality, efficiency and equity of policies, programmes and services*” ([1], p. 45) by devising delivery strategies informed by evidence about the barriers and facilitators to implementation [2, 3] in a way that explicitly considers the complexity and dynamism of real-world health systems [4, 5]. As a component of health policy and systems research [6], IR also focuses on addressing a critical missing link between research and policy/practice that permeates the public sector and impedes the use of research evidence in decision-making. In this sense, IR plays an instrumental role in informing policy-making [2], programme delivery and scale-up as well as health system strengthening more broadly. While there is increasing recognition of the importance of engaging knowledge users in implementation research [7, 8] — particularly policy-makers and decision-makers who are well-positioned to act on evidence — there is a paucity of empirical evidence on effective approaches to achieve this.

In response to the need to engage knowledge users meaningfully in research, embedded approaches to IR are gaining traction as promising approaches to optimise the delivery of health programmes and policies [9–14]. Various definitions of embedded research exist (Olivier J et al., Embedded Health Policy and Systems Research: A Rapid Scoping Review. Unpublished report for the Alliance for Health Policy and Systems Research. Geneva; 2017); for example, individual researchers embedded (physically co-located) within health service delivery settings [13, 15–17], embedding research at the organisational level [18], or institutionalising research into programme/policy processes and/or budgets [10]. There is increasing attention on embedded approaches that engage researchers and health system stakeholders — particularly those with leverage over programme/policy decision-making — in scientific inquiry, thereby integrating the research endeavour into ongoing processes of real-world programmes or policies [10, 15]. The central role of decision-makers, implementers or policy-makers in such efforts is cited as pivotal in the literature, which in addition highlights the value of collaborative research approaches that engage decision-makers alongside researchers in the co-production of evidence [7, 12, 19–22]. While there are a range of co-production models [23–27] —focusing, for example, on different agents of co-creation — a key component of research co-production is the active involvement of knowledge users, particularly decision-makers, which is increasingly recognised as an effective approach to promote the uptake of research findings. Furthermore, such approaches can reduce research waste in terms of time and effort spent on producing irrelevant or unusable evidence [28]. There is also a strong argument for integrating research endeavours within health systems and public health practice — to “*get practice into research*” ([29], p. 424) and allow evidence to be generated within its context of application [28]. Embedded research approaches are thought to address many of the barriers to research uptake documented in the literature, including the relevance and timeliness of the evidence [30, 31], while also enhancing the potential impact of research [28]. Though limited, empirical evidence suggests that such initiatives show promise in catalysing evidence-informed policy and systems decisions [13, 15].

One example of these approaches is the Improving Program Implementation through Embedded Research (iPIER) initiative jointly supported by the Pan American Health Organization (PAHO) and the Alliance for Health Policy and Services Research (the Alliance), an international partnership hosted by WHO. The objective of this initiative is to improve existing health programmes by funding IR that is conducted by teams of researchers and decision-makers, including health programme managers and directors, implementers and policy-makers. This approach ensures that health system stakeholders who are positioned to act upon research findings are integral to the entire research process from the outset, contributing to research question definition, research design and implementation as well as to the interpretation of findings to develop actionable recommendations for improvement. Through their role as co-principal investigators, decision-makers are encouraged and supported to play an influential role in the research process, fostering ownership of the evidence produced. In the context of iPIER, embedded IR was defined by the following features: situating research within ongoing health programmes/policy cycles, collaboration between researchers and programme/policy decision-makers, a primary role for decision-makers in the research endeavour, and focus on processes of programme/policy implementation and their outcomes, as is discussed in greater detail in this paper.

However, the evidence base on the processes and effectiveness of the embedded research approach remains inchoate. Systematic evaluation of such approaches is needed to advance our understanding about how they operate in diverse real-world contexts. This is particularly important given the lack of clarity about the core features and conditions of ‘embeddedness’ minimally required to achieve the objective of evidence-informed decision-making for health programme improvement. In this sense, we understood that our task of developing a systematic approach to its evaluation would require grappling with fundamental questions about the definition of embedded IR. Drawing on the extensive experience of the PAHO/Alliance iPIER project in Latin America and the Caribbean, we sought to develop a framework to guide standardised evaluations of such approaches, with the explicit aim of ensuring its applicability to a wide range of embedded research models. We developed an overarching conceptual model supported by a core set of evaluation constructs hypothesised to influence the use of research evidence by decision-makers for programme improvement. Our expectation is that the use of such a framework will be of great value in generating comparable evidence about these initiatives and contribute to identifying essential elements of ‘embeddedness’, unveiling the underlying pathways and mechanisms of change, and assessing their contribution both to evidence uptake in decision making as well as to longer-term effects on policy change or health services/systems improvements. In this paper, we present the development of this general framework^1^ for the evaluation of embedded IR approaches and provide detailed descriptions of its components with examples from previous iPIER projects to illustrate key concepts and demonstrate their broader applicability.

## Methods

### Study population: embedded research decision-makers and researchers

The iPIER initiative in Latin America and the Caribbean supported 19 IR grants to conduct embedded IR in 10 countries between 2014 and 2017. All research teams are required to include stakeholders with decision-making purview over the health programme under study, who establish partnerships with researchers. These collaborative research teams work toward the explicit aim of producing locally relevant and actionable evidence that can be used to improve the targeted programme. The research is therefore focused on issues affecting the implementation of the programme and is embedded within a specific context, set of actors and local processes intended to catalyse action in response to research findings. Each grant, approximately $30–35,000 USD in value, was implemented over a period of 12-months and received formal and structured technical assistance from an established public health institute in the region^2^ in a continuous manner to support all stages of the research. The core elements of the initiative and its rationale have been described elsewhere [10].

The majority of research teams engaged in what Aarons et al. describe as a “*first phase*” of formative evaluation to identify critical challenges and bottlenecks to implementation that subsequently inform the remedial actions proposed [33]. In this sense, most teams were engaging at the exploration phase [33], characterized by an awareness of a programme implementation issue in need of attention. A few projects focused on testing the effectiveness of new implementation strategies [34].

### The approach

The general evaluation framework presented here is the product of an iterative process of reflection and theorising that draws on several phases of evaluative work on the iPIER initiative, namely (1) a retrospective formative inquiry into the experience and scope of four previous 2014 iPIER grants, which informed an initial evaluation strategy; (2) the application of this strategy to the seven 2016 iPIER grantees through a prospective evaluation of the processes and proximal outcomes of these embedded IR projects over the 12-month funding period; (3) stakeholder consultations with content experts to vet a draft framework; and (4) an ongoing review of literature to ensure that current scholarly theorising on this topic guided and focused these efforts. Throughout, the lead author conducted analytical reflection to identify core elements of embedded IR that should be evaluated to understand whether, how and under what conditions this research approach can stimulate evidence uptake for programme improvement.

The aim of the retrospective formative inquiry was to develop both a descriptive conceptual model for the iPIER approach as well as an evaluation strategy for the 2016 grants. The draft model produced was adapted from a broader framework on embedded research by Tran et al. (“Applying a framework for embedded research to optimize the outcomes of implementation research and delivery science (IRDS) - innovations in practice,” unpublished) and further informed by a review of key project documents (i.e. research protocols, final reports or scientific manuscripts) and key informant interviews with researchers and decision-makers from four 2014 iPIER research teams (Argentina, Colombia, Mexico and Peru).

Based on this formative inquiry, a prospective process and outcomes evaluation strategy was developed and implemented on the 2016 iPIER cohort (Additional file 1) by the lead author. The purpose of this evaluation strategy was to (1) elucidate how the embedded IR model is implemented in various contexts, highlighting insights into the processes and conditions of embedded research, and (2) assess initial signals of the effectiveness of this research approach by examining proximal outcomes in the evidence co-production stage.^3^ Multiple data sources and respondent perspectives were triangulated, including 28 semi-structured key informant interviews with decision-makers, researchers and technical assistance providers, analysis of research protocols, and research team survey data. Though this exercise was based on iPIER’s specific embedded IR approach, for the purpose of developing this framework, we applied a broader analytical lens to identify a comprehensive set of constructs about embeddedness and the evidence-to-action processes involved, with the explicit aim of developing a widely applicable framework.

A final step in the development of this framework was to subject it to constructive scrutiny by experts in the field of evidence-to-action and related areas, spanning beyond public health to include public policy and political science disciplines. Convenience sampling was used to identify suitable candidates, based primarily on recommendations from the Alliance as well as on the identification of relevant scholars from our review of the literature [35]. A total of 14 experts were interviewed using a semi-structured guide to elicit informed critiques of the framework as well as recommendations to strengthen its content and structure.

An ongoing and iterative process of consulting the literature was conducted throughout to remain abreast of the rapidly evolving literature on this topic. Both database searches of key terms as well as ‘cited by’ and ‘snowball’ sampling of sources referenced in relevant articles were used [36]. To guide the literature review, we identified five broad conceptual domains that undergird the embedded IR logic (and its evaluation): (1) research impact, (2) evidence-informed decision-making, (3) research–practice partnerships (‘co-production’), (4) dissemination and implementation science, and (5) complexity theory and systems thinking for public health (Additional file 2). While this was not an exhaustive search, our analysis suggests that we began to reach saturation of key concepts and constructs for inclusion in the framework.

Throughout these various processes — both experiential and theoretical in nature — we documented priority issues for consideration in the evaluation of embedded IR approaches.

### Ethical approval

This study was submitted to the PAHO Ethical Review Committee prior to data collection. It received an exemption, deemed as not constituting research with human subjects (PAHOERC Ref. No.: 2017-02-0016). All participants were given information about the evaluation before the interviews and provided consent both to audio recording as well as to their views being reported anonymously.

## Results

**Fig. 1:**
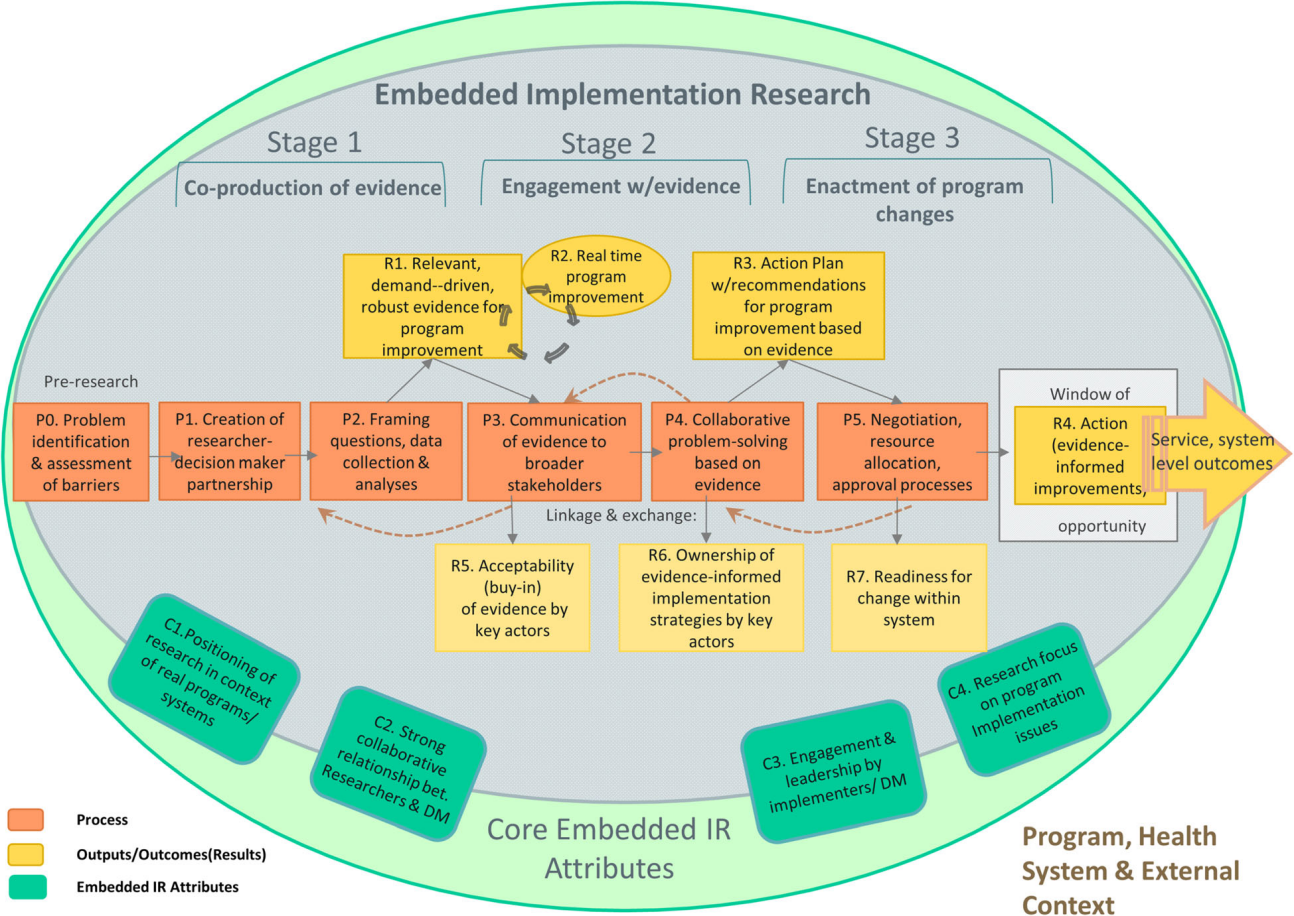
Working conceptual model for embedded implementation research. Originally adapted from Tran et al., unpublished

Through this reflection, we identified critical elements about embedded IR that could be used to assess the effectiveness (or lack thereof) of such approaches as well as the related processes and underlying influencing factors. In this section, we present our working conceptual model on embedded IR (Fig. 1) and provide an overview of the most salient evaluation constructs that underlie it.

The conceptual model for embedded IR (Fig. 1) illustrates, in broad strokes, the pathway(s) through which this approach is expected to advance the goal of evidence uptake for health programme/policy improvement. The key processes are organised along three overarching stages, as follows: (1) co-production of evidence, namely the processes related to the collaborative research endeavour itself; (2) engagement with evidence, that is the linkage and exchange processes to engage pivotal system stakeholders with the evidence; and (3) enactment of programme improvements, namely processes related to the adoption and implementation of a decision to change the programme. Depicted in the model are central elements of these processes (P0–P5), their key outputs (R5–R7) and critical incremental outcomes (R1–R4). Recognising the complexity of these processes, the inevitable overlap in stages and the expected recursive progression through phases in the process, the model underscores the iterative and non-linear nature of embedded IR. While the embedded IR endeavour may be formulated differently in distinct contexts, core attributes of embedded IR are highlighted, as follows: (C1) research is situated within an ongoing health programme/policy cycle, (C2) the research team is comprised of programme/policy decision-makers and researchers engaged in a purposeful research collaboration, (C3) active and continued engagement of the decision-makers in different stages of the research, and (C4) explicit focus of the research on programme/policy implementation issues, emphasising implementation barriers or underlying health system dysfunctions.

In an effort to develop a framework to guide the evaluation of embedded IR, we have unpacked the complexity of this overarching model, identifying numerous underlying factors that may influence embedded IR processes and therefore warrant deeper examination. We have organised these factors along four components that align with the conceptual model, namely embedded research team attributes, the key processes involved, a series of corresponding incremental outcomes, and the context in which embedded IR takes place (Additional file 3). While these components are not neatly delineated — we recognize that in the real-world some of the constructs therein may manifest themselves entangled with others — this provides a starting point to explore how these constructs influence the desired pathway of change. At this stage of development, the framework does not articulate relationships between constructs nor specific mechanisms of effect; future case study work will seek to shed light on these. The core components of the framework are described here using illustrative examples from the seven 2016 projects.

### Component 1: embedded research team attributes

This evaluation component comprises details relating to the composition and structure of the research teams as well as the mechanisms in place to facilitate collaboration within them. A critical element under this category is the specific role of the decision-maker within the research team, which may evolve and shift depending on the stage of the research process, ranging from that of a liaison or advisor to that of a leader or key motivator. In some teams, we observed decision-makers who selectively participated in specific aspects of the research, for example, in research question definition or in facilitating access to key programme resource persons. In other teams, the decision-maker assumed complete responsibility and leadership throughout the research endeavour. The decision-maker role/positioning within the health programme/system and their level of responsibility can affect their ability to act independently on findings. Issues affecting staff turnover, such as whether research team members are political appointees or the proximity of the decision-maker to programme and service implementation, are also pertinent. Decision-makers’ previous experience with research as well as researchers’ familiarity with programme implementation and improvement processes merit consideration, as does any history of professional collaboration. The affiliation of the researchers involved — whether internal or external to the public health system — may affect the relationships between researchers and decision-makers as well as the sustainability of the partnerships.

### Component 2: processes

This component refers to various key aspects of both the collaborative research itself as well as the subsequent activities to apply the research evidence for programme improvement. We therefore consider not only the research process (e.g. problem identification, framing of research questions, decision-maker participation throughout the research) and the overarching evidence-informed decision-making (EIDM) processes (e.g. linkage and exchange [37], collaborative problem-solving, action planning), but also the related interpersonal processes (e.g. establishing shared vision, team dynamics and power relations, trust-building, conflict resolution). Several research projects directly interviewed policy-makers, managers or senior decision-makers to elicit perspectives about programme implementation; this may foster credibility of the research and heighten their awareness of the need for change. Interestingly, we found that most grantees did not report on many of the challenges to collaborative research noted in the literature [38–40], such as building mutual trust, creating a shared vision for the research or conflict management; this is likely because the iPIER mechanism required grantees to self-select research partners and also to establish clear objectives, minimising such challenges. While these issues did not arise within the specific context of the iPIER initiative to date, we have included them in our framework with the aim of ensuring its applicability to a wide range of embedded research models. In terms of the involvement of decision-makers in these processes — one of the critical attributes of our conceptualisation of ‘embeddedness’ — our observations suggest that this can best be understood through a ‘continuum’ of engagement with varying levels of intensity, depending on the stage in the process, the specific team structure or the broader context.

### Component 3: outcomes

To determine whether and how embedded IR approaches are ‘effective’, we propose the assessment of a series of outcomes — proximal, intermediate and distal — aligned with the temporal logic in the hypothesised chain of effects. The intention is not to imply a linear process but rather to highlight a line of progression in the expected effects, while acknowledging the possibility for overlap and feedback loops across key stages. These effects are captured in three broad categories, namely EIDM process outcomes, implementation outcomes and health service/health system outcomes. While we believe it is important, conceptually, to identify the health outcomes ultimately targeted by implementation improvements (i.e. distal outcomes), we also recognise that direct attribution of such outcomes to the embedded IR approach is not possible and have therefore not explicitly included them in this framework.

The proximal outcomes relate to the evidence-informed decision-making processes and can serve as incremental milestones of progress, focusing on the co-production of relevant, timely evidence; dissemination of evidence and joint problem-solving with a wider group of programme stakeholders; and decisions or changes made to permit implementation of programme improvements. In cases where programme changes are not enacted, other important effects can be examined, for example, whether the IR evidence is considered in decision making, shifts in stakeholder attitudes or perspectives about the programme (due to the evidence), a decision to conduct further research to adequately solve the problem, and an informed decision not to implement any changes. Our experience demonstrates that involvement of decision-makers in IR fosters ownership of the evidence produced and heightens its responsiveness to priority information needs for programme decision-making. Furthermore, the deliberative processes that engage a wider group of programme stakeholders in dialog about how to apply the evidence may help cultivate readiness for change and foster buy-in for the solutions proposed. While the timing of the research was not always directly aligned with programme/policy processes, in some cases, the decision-makers were able to accelerate dissemination of preliminary findings to coincide with ongoing policy discussions. For example, one research team (Chile) formally presented early findings to a range of actors implicated in the policy under study, integrating the evidence into broader negotiations within the Ministry of Health about its reformulation. Other ‘soft’ outcomes, such as capacity development among non-researcher health system stakeholders (e.g. learning about research processes and acquiring research skills through their participation in the research endeavour) as well as cultivating a culture of evidence through participation in these efforts, are also key.

In cases where teams are able to introduce changes to the programme based on the IR findings, two sets of intermediate outcomes should be assessed — implementation outcomes and health services/system outcomes — across which there is admittedly some overlap. Implementation outcomes, that is acceptability, adoption, appropriateness, feasibility, fidelity, reach and sustainability [20], reflect the aspects of implementation affected by the changes introduced, which, in turn, strengthen intervention effectiveness [41], and are therefore understood as preconditions for downstream outcomes. The iPIER grants that incorporated implementation outcomes in their research tended to focus on variables relevant to earlier stages of implementation [1], examining acceptability, adoption, reach or coverage. Further downstream outcomes, stemming from the implementation outcomes produced, pertain to the effects of programme changes on health service delivery or other key health system functions, categorised according to WHO building blocks [42]. In some cases, service delivery changes may be implemented in ‘real time’ in response to the evidence as it emerges; in Colombia, the decision-maker co-principal investigator made immediate changes to address access barriers related to service hours for cervical cancer screening. The learning from previous grants suggests that changes at the service/system level should be evaluated with a longer time lag (18–24 months), depending on the type of programme intervention and the time needed to effect change.

### Component 4: context

Given the complexity of embedded IR approaches, careful consideration of the context within which they are conducted is needed to identify the factors that may affect whether and how embedded IR objectives can be reached. Aspects of ‘context’ that emerged as critical in our reflection pertain to three levels of the system and the corresponding stakeholders, namely (1) micro, the specific health programme under study; (2) meso, the health system in which the programme is embedded; and (3) macro, the broader socio-political environment. With an understanding that context is a dynamic and interacting component of implementation, it is expected that the influence of a particular contextual factor will vary in different settings, evolve over time and interact not only with the embedded IR processes but also with other factors to shift the playing field in unpredictable ways [43, 44]. While the number of potentially relevant contextual factors is extensive, some of the more prominent factors identified in our reflection include type of programme/policy under study; the perceived value of evidence in decision-making circles; political will and support for the improvement of the targeted programme (i.e. relative priority); health system governance, namely decentralisation of decision-making, staff turnover and regulatory structures or institutional incentives; political stability and underlying political interests; access to external technical assistance for IR; and availability of funding for research as well as post-research activities.

### Equity

Our framework considers health equity as a cross-cutting concept. We propose that health equity issues be explicitly addressed within the ‘process’ as well as ‘outcomes’ components [45]. The recently developed EquIR framework [46] provides a foundation for this. For example, to assess ‘process’, it proposes examining the extent to which a health equity lens is incorporated into the IR protocols or identifying changes to the programme implementation strategy that address an equity gap. Measurement of implementation outcomes [20] should also explicitly incorporate equity considerations, while further downstream, a disaggregated analysis of relevant health service/system outcomes is needed to assess whether ultimately these are equitably distributed — this may focus on differences by geographic area, gender, race/ethnic group, wealth status or other indicators of vulnerability.

## Discussion

### The Embedded Implementation Research Evaluation Framework

Our framework responds to the need for rigorous and systematic evaluation of embedded IR that can expand the evidence base about the processes and effectiveness of such models in influencing programme/policy decision-making. While our structured reflection was based on the iPIER approach, we used a broad analytical lens to identify generic features and constructs of embedded IR, resulting in a widely applicable evaluation framework. Intended to serve as a heuristic tool to guide the design of evaluations on embedded IR models, the framework presents the expected processes of change for embedded IR, which we have unpacked by specifying key constructs that underlie this theory. Drawing from our real-world experience, we recognise the complexity of embedded IR endeavours and have therefore sought to capture a wide range of factors that can be studied to uncover the drivers and mechanisms of change in different settings. The framework is intentionally comprehensive, allowing for flexibility and adaptation to diverse models and contexts. We believe the framework proposed here is a significant contribution to the field as it moves beyond an evaluation of effectiveness — assessing ‘did it work’ — to examine why it was (or was not) effective in catalysing the use of evidence to inform health programme changes, while also considering how and under what circumstances it was effective (or ineffective) [47].

To the best of our knowledge, no such ‘meta’ framework exists that specifically aims to guide the evaluation of embedded IR approaches in terms of both the related processes as well as the outcomes along the expected pathway(s) of change. Numerous conceptual frameworks in the literature (or theory-building pieces) address distinct dimensions of the embedded IR model, for example, research impact or evaluation of research [27, 48–53], research utilisation and evidence-informed decision-making [2, 54–63], collaborative research–practice partnerships [12, 19, 39, 40, 64–66], dissemination and implementation science [1, 20, 41, 67–69], and complexity theory and systems thinking in public health [5, 43, 70–73]. However, no single framework or conceptual piece takes a comprehensive look at all of these core dimensions of embedded IR for the purpose of its evaluation. Our framework seeks to interweave elements from these various conceptual areas, thereby enabling a holistic understanding of embedded IR. In this sense, the novelty of the framework is not in presenting a new theory but rather in drawing from real-world experience to bring together key concepts from a range of theories.

The comprehensive nature of the framework requires evaluators to identify at the outset the dimensions and attributes relevant for the specific embedded research approach and setting, while remaining attentive to developments that may warrant inclusion of other evaluation constructs in subsequent stages. We have deliberately not indicated the priority level of any constructs to permit the evaluator to do so according to local context and the specific characteristics of each approach. This demands thoughtful and informed consideration of the factors most salient for each evaluation effort, with emphasis on those factors that are more amenable to change [74].

### Advancing a fundamental question: what is embedded research?

Our structured reflection highlighted two key related but distinct dimensions of embeddedness, namely (1) active engagement of decision-makers, practitioners or policy-makers in the research and (2) the positionality of these actors with respect to programme/policy decision-making processes, both of which can be conceived of in myriad forms. While there is no consensus yet in the literature as to how to embed research, who is being embedded, where and through what mechanisms, several recent efforts have sought to address these questions [10, 13, 15, 75], developing diverse conceptualisations — each emphasising certain features over others or targeting different stakeholders (e.g. service users versus decision-makers). Given the limited empirical evidence on the specific aspects of embedded IR that are most effective, we hesitated to set rigid boundaries on what is to be considered ‘embedded’. Instead, we propose various constructs that can be combined to define ‘embeddedness’.

Several important questions about ‘embeddedness’ remain. Is it possible to identify certain minimum criteria of embeddedness — or minimum ‘levels’ of a criterion — necessary for effective co-production and uptake of research for programme improvement? It is likely that some features are not static but instead will vary according to the stage of the process. For example, the involvement of decision-makers may shift in intensity from one stage to another, depending on the task at hand and their capacity to contribute to it. This raises the notion of a ‘continuum’ of embeddedness, similar to that described (and debated) by other authors [4, 27, 76, 77], suggesting that the extent to which a particular embedded IR model ‘fulfils’ a single criterion may vary along a spectrum. Additionally, questions arise as to whether there are specific circumstances under which certain aspects of ‘embeddedness’ are more pivotal than others — what are the optimal conditions for embedding research? This paper lays the groundwork for exploring such questions and complements ongoing theoretical reflections on embeddedness in and beyond the health sector. Forthcoming research by the lead author will apply this framework through case studies of iPIER embedded IR projects to examine such questions in greater depth as well as to validate the framework [78]. We call on others to explore similar questions, applying this framework to diverse embedded research initiatives with a view not only to further build the evidence about embedded IR but also to assess the framework’s utility and relevance, thereby contributing to refining and strengthening it.

### Institutionalisation: an area for further reflection

It is important to acknowledge that notably missing from this framework are issues around institutionalisation, both in terms of sustaining the improvements made to the programme (based on the evidence) as well as in formalising and routinising embedded research into programme processes. The former relates to the scale-up and sustainability of changes made to the programme implementation strategy and is the focus of an entire body of literature [79–83]; the latter falls under the umbrella of creation (and maintenance) of health research systems [84–87]. While we understand these to be essential components of the broader pathway to strengthened health systems and improved population health outcomes, they were deemed outside the focus of our immediate evaluation aims. We recognise, however, that many factors in the early stages of embedded IR may drive or influence later stage processes of institutionalisation. We expect that some evaluation constructs included in our framework are likely to influence the ability to sustain or institutionalise this research approach but their effects need to be studied explicitly. Our hope is that future iterations of this framework will be informed by research on such effects.

### Limitations and challenges

While this structured reflection sought to systematically examine and extract insights from the real-world experience of the iPIER embedded research grants, a few limitations related to our approach are worth noting. First, though this framework is based on real-world experience, not all of its components and sub-components have been applied to real-world cases and therefore require further empirical work to confirm their relevance/utility and to fully operationalise constructs. Second, due to the nature of the 12-month grant timeframe, the application of this framework was limited to the evidence co-production stage of the conceptual model. Therefore, we were not able to empirically analyse the intermediate or distal outcomes among the 2016 projects; these were considered retrospectively for prior grants. Third, several concepts in this framework, including collaboration, engagement, leadership, etc., are considered ‘soft’, intangible constructs and are therefore difficult to measure in a reliable and valid manner. There is considerable work remaining to develop and validate appropriate assessment methods for such constructs. Fourth, our review of the literature was not exhaustive. It is possible that other related frameworks or approaches, particularly those outside the field of health, could have contributed to the development of our framework. Finally, as this is a newly developed framework, further application to embedded IR projects across diverse contexts is needed to validate key concepts and the underlying logic as well as to assess its utility and relevance for evaluating embedded IR.

Furthermore, it is important to acknowledge that the embedded IR approach itself may also present certain limitations, or potential pitfalls, that must be guarded against or, at a minimum, should be deliberately considered. Numerous trade-offs need to be weighed in deciding whether or not to ‘embed’ research [88, 89]. Several sources of bias may result from the programme decision-maker’s participation in scientific inquiry, the most obvious of which is the potential for loss of objectivity in the investigation that may arise from the direct influence or pressures of programme decision-makers [28]. Biases may also be introduced in other aspects of the research, including bias in the identification of the research problem to be addressed, in respondent selection, or in whether to involve other influential stakeholders in key decisions about the research, among others. Issues may also arise in holding key actors accountable for the remedial measures emanating from study findings. The “*blurring*” of research and practice roles and activities ([90], p. 173) complicates these issues further. Like other co-production models, embedded IR is by no means a “*panacea*” ([38], p. 222) and may not always be the most appropriate approach.

## Conclusions

Given the unique — and potentially impactful — role the embedded IR approach proposes for decision-makers in the production and use of research for programme and policy improvement, there is great value in advancing rigorous strategies for its evaluation. Our evaluation framework moves a step in this direction, providing a comprehensive, broadly applicable structure through which to assess a range of embedded research initiatives. Application of this framework should occur from the outset of the embedded research process to allow for prospective and explicit examination of key driving factors. Learning generated from further application of this framework can help disentangle the various factors at play and tease out the mechanisms through which these initiatives can stimulate the use of evidence for programme/policy improvement in low- and middle-income settings. By systematising evaluation efforts, the use of this framework can create opportunities for comparison of different approaches, thereby advancing the science and practice of embedded research. Other possible uses include guidance in the design of new embedded research approaches and guidance for reporting on evaluations of embedded research as well as a tool to develop calls for grant applications or to focus research proposals. Given the potential of this embedded IR approach to bridge implementation gaps for critical health policies and interventions, systematic evaluation efforts, such as we propose here, are essential to understand the contribution to system level objectives, e.g. universal health coverage or the Sustainable Development Goals, and to cultivate the “*learning health systems*” ([91], p. xv–xvi) that these objectives require [92].

## Supplementary information

**Additional file 1.** Embedded IR Project Summaries. Overview information about the seven embedded IR projects considered in the development of the evaluation framework, including the health programme, research team member affiliations, general research objective, primary research question, research design/methods, and geographic scope of the investigation.

**Additional file 2.** Literature that informed the development of the evaluation framework. List of articles reviewed during the development of the evaluation framework, organised according to five domains — research impact, research utilisation and EIDM, research collaboration/research–practice partnerships, implementation research/dissemination and implementation science, complexity theory and systems thinking.

**Additional file 3.** Summary table of key evaluation constructs underlying embedded IR conceptual framework, organised along the domains of Embedded IR Attributes, Processes, Outcomes and Context.

## Data Availability

Data sharing is not applicable to this article as no datasets were generated or analysed during the current study.

## Abbreviations

EIDM: Evidence-informed decision-making
iPIER: Improving Program Implementation through Embedded Research
IR: Implementation research
PAHO: Pan American Health Organization

## Footnotes

1 We use ‘framework’, as defined by in Bauer et al. [32] “*Frameworks provide a broad set of constructs that organize concepts and data descriptively without specifying causal relationships*.”

2 For the 2014/2015 grants, support was provided by the Institute for Clinical Effectiveness and Health (*Instituto de Efectividad Clinica y Sanitaria*—IECS) in Argentina; for 2016/2017, this was provided by the *Instituto National de Salud Publica* of Mexico.

3 Application of the framework beyond this stage was not possible, as the iPIER projects had not yet advanced to subsequent stages.
