# Supplementary material for: Health system decision-makers at the helm of implementation research: development of a framework to evaluate the processes and effectiveness of embedded approaches
Source: Health Res Policy Syst. 2020 Jun 10;18:64. doi: 10.1186/s12961-020-00579-9 (PMC7288439; doi:10.1186/s12961-020-00579-9)
Supplement: Supplementary file 1 — Additional file 1. Embedded IR Project Summaries. Overview information about the seven embedded IR projects considered in the development of the evaluation framework, including the health programme, research team member affiliations, general research objective, primary research question, research design/methods, and geographic scope of the investigation. [file 12961_2020_579_MOESM1_ESM.docx]

**Additional File 1: Summary Table of 2016/2017 iPIER Embedded IR Projects**

| **Targeted Program** | **Decision Maker Affiliation** | **Researcher Affiliation** | **General Objective** | **Research Question** | **Research design/ Methods** | **Geographic Scope of investigation** |
| --- | --- | --- | --- | --- | --- | --- |
| **Argentina** |  |  |  |  |  |  |
| TB Control and Prevention Program in Penitentiary System | Provincial Ministry of Health Director and Sub-secretariat for health among Incarcerated Populations | Non-profit social sciences research Institute (CEDES) | Identify the main barriers and facilitators in the implementation of the TB prevention and control program for incarcerated populations in the Province of Buenos Aires since the establishment of an inter-ministerial agreement (2009) | How were the strategies for the prevention and control of TB in the incarcerated populations in PBA implemented, and what has been its acceptability, adherence and adoption by the Penitentiary Health Service? How could the implementation strategy of the TB program be modified in the PBA in order to favor its adoption by the Provincial Penitentiary Health Service? | Qualitative—Key informant interviews at multiple levels of penitentiary health system | Provincial |
| **Bolivia** |  |  |  |  |  |  |
| The C*hispitas* micronutrient supplement strategy for children | Regional coordinator of the municipal health network | Researcher and pediatrician at the municipal hospital | To understand, in the context of the “Zero Malnutrition” Program, the barriers and facilitators in adherence to *Chispitas* nutritional supplements in children between 6 and 23 months of age in Los Andes health network and design an action plan for its improvement. | What are the elements of the program that affected adherence to the consumption of *Chispitas* nutritional supplements in children between 6 and 23 months of age and how could adherence be improved? | Qualitative—Key informant interviews, direct observation of service delivery, and focus group discussions. | Local—Municipal/Health Network |
| **Brazil** |  |  |  |  |  |  |
| Psychosocial Care Program (Mental Health) | Mental Health Services of the Federal District Health Department | National research institute (Fiocruz) | Identify the main barriers and facilitators to implementation of actions and strategies in order to strengthen the role of and guarantee the rights of users in Psychosocial Care Centers | What are the actions and strategies carried out by Psychosocial Care Centers that strengthen the role of and guarantee users' rights and how are they implemented? | Qualitative—key informant interviews, focus group discussions, direct observation of service delivery, and document review. | Regional—State level (Distrito Federal de Brasilia) |
| **Chile** |  |  |  |  |  |  |
| Policy on Interchangeability of Pharmaceuticals | MOH/Central-Pharmaceutical policies division | MOH/Central planning division | Study the impact of the drug interchangeability policy and identify the processes and mechanisms of its implementation, which have generated results opposite to those proposed, in order to prepare recommendations for its reorientation. | Why has the policy on drug interchangeability not had the expected results and what should be done to make it an initiative that improves access to quality medicines for the population? | Mixed methods—Quantitative: interrupted time series analysis of impact of current generic drug substitution policy; Qualitative: key informant interviews | National |
| **Colombia** |  |  |  |  |  |  |
| Program for cervical cancer screening | Municipal health system manager | Independent research consultants | Identify strategies related to access and quality of care within the health services in the network of public facilities in Cali that affect the levels of vaginal cytology coverage; based on this, make suggestions for improvement. | How do the strategies related to access and quality of care, used by the public health services of Cali, affect the levels of vaginal cytology coverage in the city? | Qualitative—Key informant interviews, direct observations of service delivery, document review, focus group discussions | Local—Municipal |
| **Dominican Republic** | **c** |  |  |  |  |  |
| Family Planning Program, Men’s Health Strategy | MOH-Office of Gender Equity and Development | Academic research institute | Identify the mechanisms that facilitate the effective integration of men as a beneficiary population in the implementation of the Family Planning Program. | Has the absence of a gender focus in the design and implementation of the Family Planning Program been an obstacle to the integration of men as active subjects in contraception? | Qualitative—Key informant interviews, focus group discussions, and document review. | National |
| **Peru** |  |  |  |  |  |  |
| TB Control and Prevention Program | Regional Health Directorate | National Institute of Health (INS) - Unit on Public Health Evidence Analysis and Generation | Identify the factors influencing the implementation of the Regional Health Strategy for Prevention and Control of TB in primary care that influence adherence to treatment from the perspective of the provider and the user. | What factors in the implementation of the TB program affect adherence to treatment from the perspective of the provider and the user? | Mixed—Qualitative interviews and focus group discussions; Quantitative health facility survey. | Regional |
