# Supplementary material for: Health system decision-makers at the helm of implementation research: development of a framework to evaluate the processes and effectiveness of embedded approaches
Source: Health Res Policy Syst. 2020 Jun 10;18:64. doi: 10.1186/s12961-020-00579-9 (PMC7288439; doi:10.1186/s12961-020-00579-9)
Supplement: Supplementary file 2 — Additional file 2. Literature that informed the development of the evaluation framework. List of articles reviewed during the development of the evaluation framework, organised according to five domains — research impact, research utilisation and EIDM, research collaboration/research–practice partnerships, implementation research/dissemination and implementation science, complexity theory and systems thinking. [file 12961_2020_579_MOESM2_ESM.docx]

| **Additional File 2: Literature referenced in development of evaluation framework for embedded implementation research** | | | |
| --- | --- | --- | --- |
|  | | |  |
|  | | |  |
| **Research Impact** | | | |
|  | | Banzi, R., Moja, L., Pistotti, V., Facchini, A., & Liberati, A. (2011). Conceptual frameworks and empirical approaches used to assess the impact of health research: an overview of reviews. Health Research Policy and Systems, 9(1), 26. | |
|  | | Boaz, A., Fitzpatrick, S., & Shaw, B. (2009). Assessing the impact of research on policy: a literature review. Science and Public Policy, 36(4), 255-270. | |
|  | | Greenhalgh, T., Robert, G., Macfarlane, F., Bate, P., & Kyriakidou, O. (2004). Diffusion of innovations in service organizations: systematic review and recommendations. The Milbank Quarterly, 82(4), 581-629. | |
|  | | Greenhalgh, T., Jackson, C., Shaw, S., & Janamian, T. (2016). Achieving research impact through co‐creation in community‐based health services: literature review and case study. The Milbank Quarterly, 94(2), 392-429. | |
|  | | Hanney, S., Greenhalgh, T., Blatch-Jones, A., Glover, M., & Raftery, J. (2017). The impact on healthcare, policy and practice from 36 multi-project research programmes: findings from two reviews. Health Research Policy and Systems, 15(1), 26. | |
|  | | Kuruvilla S. et al. (2006) ‘Describing the Impact of Health Research: A Research Impact Framework’, BMC Health Services Research , 6: 134. | |
|  | Lavis, J., Ross, S., McLeod, C., & Gildiner, A. (2003). Measuring the impact of health research. Journal of Health Services Research & Policy, 8(3), 165-170. | | |
|  | | Morton, S. (2015). Progressing research impact assessment: A ‘contributions’ approach. Research Evaluation, 24(4), 405-419. | |

| **Research Utilization and Evidence-informed Decision Making (EIDM)** | |
| --- | --- |
| *Embedded Research* | |
|  | Ghaffar, A., Langlois, E. V., Rasanathan, K., Peterson, S., Adedokun, L., & Tran, N. T. (2017). Strengthening health systems through embedded research. Bulletin of the World Health Organization, 95(2), 87. |
|  | Koon, A. D., Rao, K. D., Tran, N. T., & Ghaffar, A. (2013). Embedding health policy and systems research into decision-making processes in low-and middle-income countries.Health Research Policy and Systems, 11(1), 30. |
|  | Langlois, et al., forthcoming. Embedded implementation research to enhance health policy and systems: a multi-country analysis from eight Latin American and Caribbean countries. Bulletin of the World Health Organization. |
|  | Olivier, J., Whyle, E., Gilson, L. (unpublished). Embedded Health Policy and Systems Research: Rapid Scoping Review. Report for The Alliance for Health Policy and Systems Research, World Health Organization. |
|  | Tran, N., Langlois, E. V., Reveiz, L., Varallyay, I., Elias, V., Mancuso, A., ... & Ghaffar, A. (2017). Embedding research to improve program implementation in Latin America and the Caribbean. Revista Panamericana de Salud Pública, 41. |
|  | Vindrola-Padros, C., Pape, T., Utley, M., & Fulop, N. J. (2017). The role of embedded research in quality improvement: a narrative review. BMJ Qual Saf, 26(1), 70-80. |
| *Knowledge-to-action/ Evidence-to-policy* | |
|  | Andermann, A., Pang, T., Newton, J. N., Davis, A., & Panisset, U. (2016). Evidence for Health I: Producing evidence for improving health and reducing inequities. Health Research Policy and Systems, 14(1), 1. |
|  | Aarons, G. A., Hurlburt, M., & Horwitz, S. M. (2011). Advancing a conceptual model of evidence-based practice implementation in public service sectors. Administration and Policy in Mental Health and Mental Health Services Research, 38(1), 4-23. |
|  | Bhattacharyya, O. K., Estey, E. A., & Zwarenstein, M. (2011). Methodologies to evaluate the effectiveness of knowledge translation interventions: a primer for researchers and health care managers. Journal of Clinical Epidemiology, 64(1), 32-40. |
|  | Bowen, S., & Zwi, A. B. (2005). Pathways to “evidence-informed” policy and practice: a framework for action. PLoS Medicine, 2(7), e166. |
|  | Chambers, D. A., Glasgow, R. E., & Stange, K. C. (2013). The dynamic sustainability framework: addressing the paradox of sustainment amid ongoing change. Implementation Science, 8(1), 117. |
|  | Contandriopoulos, D., Lemire, M., Denis, J. L., & Tremblay, É. (2010). Knowledge exchange processes in organizations and policy arenas: a narrative systematic review of the literature. The Milbank Quarterly, 88(4), 444-483. |
|  | Davison, C. M., Ndumbe-Eyoh, S., & Clement, C. (2015). Critical examination of knowledge to action models and implications for promoting health equity. International Journal for Equity in Health, 14(1), 49. |
|  | Gagliardi, A. R., Berta, W., Kothari, A., Boyko, J., & Urquhart, R. (2015). Integrated knowledge translation (IKT) in health care: a scoping review. Implementation Science, 11(1), 38. |
|  | Graham, I.D., Logan, J., Harrison, M.B., Straus, S.E., Tetroe, J., & Caswell,W (2006). Lost in knowledge translation: Time for a map? The Journal of Continuing Education in the Health Professions, 26, 13–24 |
|  | Grimshaw, J. M., Eccles, M. P., Lavis, J. N., Hill, S. J., & Squires, J. E. (2012). Knowledge translation of research findings. Implementation science, 7(1), 50. |
|  | Jacobson, N., Butterill, D., & Goering, P. (2003). Development of a framework for knowledge translation: understanding user context. Journal of Health Services Research & Policy, 8(2), 94-99. |
|  | Mitton, C., Adair, C. E., McKenzie, E., Patten, S. B., & Perry, B. W. (2007). Knowledge transfer and exchange: review and synthesis of the literature. The Milbank Quarterly, 85(4), 729-768. |
|  | Nutley, S., Walter, I., & Davies, H. T. (2003). From knowing to doing: a framework for understanding the evidence-into-practice agenda. Evaluation, 9(2), 125-148. |
|  | Panisset, U., Koehlmoos, T. P., Alkhatib, A. H., Pantoja, T., Singh, P., Kengey-Kayondo, J., ... & Miguel, G. B. Á. (2012). Implementation research evidence uptake and use for policy-making. Health Research Policy and Systems, 10(1), 20. |
|  | Sarkies, M. N., Bowles, K. A., Skinner, E. H., Haas, R., Lane, H., & Haines, T. P. (2017). The effectiveness of research implementation strategies for promoting evidence-informed policy and management decisions in healthcare: a systematic review. Implementation Science, 12(1), 132. |
|  | Siron, S., Dagenais, C., & Ridde, V. (2015). What research tells us about knowledge transfer strategies to improve public health in low-income countries: a scoping review. International journal of Public Health, 60(7), 849-863. |
|  | Ward, V., Smith, S., House, A., & Hamer, S. (2012). Exploring knowledge exchange: a useful framework for practice and policy. Social Science & Medicine, 74(3), 297-304. |
|  | Woolf, S. H., Purnell, J. Q., Simon, S. M., Zimmerman, E. B., Camberos, G. J., Haley, A., & Fields, R. P. (2015). Translating evidence into population health improvement: strategies and barriers. Annual Review of Public Health, 36, 463-482. |
|  | Van Eerd, D., Cole, D., Keown, K., Irvin, E., Kramer, D., Brenneman Gibson, J., ... & Phipps, D. (2011). Report on knowledge transfer and exchange practices: a systematic review of the quality and types of instruments used to assess KTE implementation and impact. Toronto: Institute for Work & Health. |
| *Research Utilization* | |
|  | Hanney, S. R., Gonzalez-Block, M. A., Buxton, M. J., & Kogan, M. (2003). The utilisation of health research in policy-making: concepts, examples and methods of assessment. Health Research Policy and Systems, 1(1), 2. |
|  | Kim, C., Wilcher, R., Petruney, T., Krueger, K., Wynne, L., & Zan, T. (2018). A research utilisation framework for informing global health and development policies and programmes. Health Research Policy and Systems, 16(1), 9. |
|  | Kok, M. O., Gyapong, J. O., Wolffers, I., Ofori-Adjei, D., & Ruitenberg, J. (2016). Which health research gets used and why? An empirical analysis of 30 cases. Health Research Policy and Systems, 14(1), 36. |
|  | Liverani, M., Hawkins, B., & Parkhurst, J. O. (2013). Political and institutional influences on the use of evidence in public health policy. A systematic review. PLoS One, 8(10), e77404. |
|  | Moore, G., Redman, S., Haines, M., & Todd, A. (2011). What works to increase the use of research in population health policy and programmes: a review. Evidence & Policy: A Journal of Research, Debate and Practice, 7(3), 277-305. |
|  | Oliver, K., Innvar, S., Lorenc, T., Woodman, J., & Thomas, J. (2014). A systematic review of barriers to and facilitators of the use of evidence by policymakers. BMC Health Services Research, 14(1). |
|  | Orton, L., Lloyd-Williams, F., Taylor-Robinson, D., O'Flaherty, M., & Capewell, S. (2011). The use of research evidence in public health decision making processes: systematic review. PLoS One, 6(7), e21704. |
|  | Redman, S., Turner, T., Davies, H., Williamson, A., Haynes, A., Brennan, S., ... & Green, S. (2015). The SPIRIT Action Framework: A structured approach to selecting and testing strategies to increase the use of research in policy. Social Science & Medicine, 136, 147-155. |

| **Research Collaboration/ Research-Practice Partnerships** | |  |
| --- | --- | --- |
|  | Brinkerhoff, J. M. (2002). Assessing and improving partnership relationships and outcomes: a proposed framework. Evaluation and Program Planning, 25, 215–131. | |
|  | Bullock, A., Morris, Z. S., & Atwell, C. (2012). Collaboration between health services managers and researchers: making a difference?. Journal of Health Services Research & Policy, 17(2_suppl), 2-10. | |
|  | Heaton, J., Day, J., & Britten, N. (2016). Collaborative research and the co-production of knowledge for practice: an illustrative case study. Implementation Science, 11(1), 20. | |
|  | Kothari, A., MacLean, L., Edwards, N., & Hobbs, A. (2011). Indicators at the interface: managing policymaker-researcher collaboration. Knowledge Management Research & Practice, 9(3), 203-214. | |
|  | Lomas, J. (2000). Using 'linkage and exchange 'to move research into policy at a Canadian foundation. Health Affairs, 19(3), 236-240. | |
|  | Mitchell, P., Pirkis, J., Hall, J., & Haas, M. (2009). Partnerships for knowledge exchange in health services research, policy and practice. Journal of Health Services Research & Policy, 14(2), 104-111. | |
|  | Ross, S., Lavis, J., Rodriguez, C., Woodside, J., & Denis, J. L. (2003). Partnership experiences: involving decision-makers in the research process. Journal of Health Services Research & Policy, 8(2_suppl), 26-34. | |
|  | Sturke, R., Siberry, G., Mofenson, L., Watts, D. H., McIntyre, J. A., Brouwers, P., & Guay, L. (2016). Creating sustainable collaborations for implementation science: the case of the NIH-PEPFAR PMTCT Implementation Science Alliance. JAIDS Journal of Acquired Immune Deficiency Syndromes, 72, S102-S107. | |
|  | Traynor, R., Dobbins, M., & DeCorby, K. (2015). Challenges of partnership research: insights from a collaborative partnership in evidence-informed public health decision making. Evidence & Policy: A Journal of Research, Debate and Practice, 11(1), 99-109. | |
|  | Wehrens, R. (2014). Beyond two communities–from research utilization and knowledge translation to co-production?. Public Health, 128(6), 545-551. | |

| **Implementation Research/ Dissemination & Implementation Science** | |  |
| --- | --- | --- |
|  | Damschroder, L. J., Aron, D. C., Keith, R. E., Kirsh, S. R., Alexander, J. A., & Lowery, J. C. (2009). Fostering implementation of health services research findings into practice: a consolidated framework for advancing implementation science. Implementation science, 4(1), 50. | |
|  | Fixsen, D. L., Naoom, S. F., Blase, K. A., & Friedman, R. M. (2005). Implementation research: a synthesis of the literature. Tampa: University of South Florida, Louis de la Parte Florida Mental Health Institute, The National Implementation Research Network. | |
|  | Nilsen, P. (2015). Making sense of implementation theories, models and frameworks. Implementation Science, 10(1), 53. | |
|  | Panisset, U., Koehlmoos, T. P., Alkhatib, A. H., Pantoja, T., Singh, P., Kengey-Kayondo, J., ... & Miguel, G. B. Á. (2012). Implementation research evidence uptake and use for policy-making. Health Research Policy and Systems, 10(1), 20. | |
|  | Peters D, Adams T, Alonge O, Agyepong I, and Tran N (2013). Implementation Research: What it is and how to do it. BMJ 2013; 347: f6753. | |
|  | Proctor, E., Silmere, H., Raghavan, R., Hovmand, P., Aarons, G., Bunger, A., ... & Hensley, M. (2011). Outcomes for implementation research: conceptual distinctions, measurement challenges, and research agenda. Administration and Policy in Mental Health and Mental Health Services Research, 38(2), 65-76. | |
|  | Skolarus, T. A., Lehmann, T., Tabak, R. G., Harris, J., Lecy, J., & Sales, A. E. (2017). Assessing citation networks for dissemination and implementation research frameworks. Implementation Science, 12(1), 97. | |
|  | Tabak, R. G., Khoong, E. C., Chambers, D. A., & Brownson, R. C. (2012). Bridging research and practice: models for dissemination and implementation research. American Journal of Preventive Medicine, 43(3), 337-350. | |
|  | Moullin, J. C., Sabater-Hernández, D., Fernandez-Llimos, F., & Benrimoj, S. I. (2015). A systematic review of implementation frameworks of innovations in healthcare and resulting generic implementation framework. Health research policy and systems, 13(1), 16. | |

| **Complexity Theory and Systems Thinking** | |
| --- | --- |
|  | Best, A., & Holmes, B. (2010). Systems thinking, knowledge and action: towards better models and methods. Evidence & Policy: A Journal of Research, Debate and Practice, 6(2), 145-159. |
|  | Burke, J. G., Lich, K. H., Neal, J. W., Meissner, H. I., Yonas, M., & Mabry, P. L. (2015). Enhancing dissemination and implementation research using systems science methods. International Journal of Behavioral Medicine, 22(3), 283-291. |
|  | Cherney, Adrian, and Brian W. Head. (2011). Supporting the Knowledge to Action Process: A Systems-Thinking Approach. Evidence & Policy 7(4): 471–88. |
|  | Hawe, P. (2015). Lessons from Complex Interventions to Improve Health. Annu. Rev. Public Health, 36, 307–23. |
|  | Holmes BJ, Finegood DT, Riley BL, Best A. (2012). Systems thinking in dissemination and implementation research. In: Brownson RC, Colditz GA, Proctor EK, editors. Dissemination and Implementation Research in Health: Translating Science to Practice. New York: Oxford University Press; p. 175–191. |
|  | Pfadenhauer, L. M., Gerhardus, A., Mozygemba, K., Lysdahl, K. B., Booth, A., Hofmann, B., ... & Rehfuess, E. (2017). Making sense of complexity in context and implementation: the Context and Implementation of Complex Interventions (CICI) framework. Implementation Science, 12(1), 21. |
