# Supplementary material for: Health system decision-makers at the helm of implementation research: development of a framework to evaluate the processes and effectiveness of embedded approaches
Source: Health Res Policy Syst. 2020 Jun 10;18:64. doi: 10.1186/s12961-020-00579-9 (PMC7288439; doi:10.1186/s12961-020-00579-9)
Supplement: Supplementary file 3 — Additional file 3. Summary table of key evaluation constructs underlying embedded IR conceptual framework, organised along the domains of Embedded IR Attributes, Processes, Outcomes and Context. [file 12961_2020_579_MOESM3_ESM.docx]

**Additional File 3: Summary table of key evaluation constructs underlying embedded IR conceptual framework, organized along domains: Embedded IR Attributes, Processes, Outcomes, and Context**

| Embedded IR Attributes |
| --- |
| - Decision maker role in research |
| - Decision maker positioning in program/system (type of decision maker, level of authority) |
| - Research capacity and absorptive capacity of decision makers |
| - Researcher affiliation (internal/external to Ministry of Health) |
| - Partnership mechanisms and governance structures |
| - Prior history of collaboration between program decision maker and researcher |

- Mutual trust between researcher and decision maker at outset

| Processes |  |
| --- | --- |
| Research Process |  |
| - Source of initial impetus to conduct IR (problem identification) |  |
| - Focusing research on ‘implementation’ (framing research questions) |  |
| - Decision maker participation & intensity of engagement |  |
| - Surfacing and sharing tacit knowledge (values, mindsets, experiences) |  |
| - Facilitation/support (eg. incentives, technical support, capacity development) |  |
| Interpersonal Processes |  |
| - Shared understanding/vision |  |
| - Trust building |  |
| - Power dynamics; power-sharing and co-governance |  |
| - Mutual benefit; incentives |  |
| - Collaborative team work |  |
| - Conflict resolution (“productive conflict”) |  |
| Evidence-informed Decision Making Processes |  |
| - Linkage and exchange; continued engagement beyond research phase |  |
| - Deliberation and collaborative problem-solving |  |
| - Strategizing and action planning |  |
| - Negotiation and approvals (resource allocation) |  |
| - Preparations and health system adjustments for change |  |
| Outcomes | |
| Process outputs and proximal outcomes | |
| - Relevant, timely, interpretable, and credible IR findings | |
| - IR evidence considered in problem-solving | |
| - Actionable recommendations for program improvement | |
| - Ownership of proposed program improvements by decision makers | |
| - Readiness for change within system | |
| - Adoption decision and implementation of program changes (including ‘real time’ changes) | |
| - Capacity development (both researchers and decision makers) | |
| - Cultivating a culture of evidence | |
| Implementation outcomes [intermediate] | |
| - Acceptability, Feasibility, Appropriateness/fit | |
| - Adoption, Fidelity, Cost | |
| - Reach, Sustainability | |
| Health service/system outcomes [intermediate] | |
| - Access, Utilization, Adherence | |
| - Service delivery quality & Patient satisfaction | |
| - Resource allocation, Efficiency | |
| - Health equity, cross-cutting through all stages | |

| Context |
| --- |
| Program Context |
| - Nature of health program under study (e.g. complexity of the program, degree of public sensitivity around program, type of program/intervention, stage of implementation, etc.) |
| - Significance of research questions relative to other program priorities (issue prioritization) |
| - Openness to change among program stakeholders |
| - Availability of other relevant information to inform program decisions (eg. routine monitoring data; political pressure; experiential knowledge/ colleague’s opinions; other research findings) |
| - Organizational structures, particularly those that relate to program decision making |
| - Availability of resources/support (financial, HR, infrastructure) to implement large scale change |
| Health System Context |
| - Capacity of the health system to adapt to change |
| - Health program prioritization within broader public health agenda |
| - Health system governance and decision making structures; e.g. degree of decentralization in decision making |
| - Other parallel initiatives that could divert attention/resources from program improvement processes |
| - Political climate around research issue (goodwill and receptivity to change) |
| - Perceptions of importance/value of implementation research |
| - Incentives or institutional arrangements that facilitate the use of research evidence for decision making (e.g. mandates on role of evidence for program improvement, national health research systems, or other research platforms) |
| External Context |
| - External forces and interests (e.g. pharmaceutical industry, professional associations, religious groups, public opinion, donors, other third party actors advocating for program changes) |
| - Demand for evidence-informed interventions (value placed on research evidence) |
| - Political stability & stage in election cycle |
| - Broader political economy |
